# Physiological responses and adaptations to high methane production in Japanese Black cattle

**DOI:** 10.1038/s41598-022-15146-1

**Published:** 2022-07-01

**Authors:** Minji Kim, Tatsunori Masaki, Kentaro Ikuta, Eiji Iwamoto, Koki Nishihara, Makoto Hirai, Yoshinobu Uemoto, Fuminori Terada, Sanggun Roh

**Affiliations:** 1 Graduate School of Agricultural Science, Tohoku University, Sendai, 980-8572 Japan; 2 Hyogo Prefectural Technology Center of Agriculture, Forestry and Fisheries, Kasai, Hyogo 679-0198 Japan; 3 Present Address: Central Research Laboratories, Nippon Zenyaku Kogyo Co., Ltd., Koriyama, Fukushima 963-0196 Japan; 4 Present Address: National Institute of Livestock and Grassland Science, National Agriculture and Food Research Organization, Ikenodai, Tsukuba, 305-0901 Japan

**Keywords:** Animal physiology, Metabolomics

## Abstract

In this study, using enteric methane emissions, we investigated the metabolic characteristics of Japanese Black cattle. Their methane emissions were measured at early (age 13 months), middle (20 months), and late fattening phases (28 months). Cattle with the highest and lowest methane emissions were selected based on the residual methane emission values, and their liver transcriptome, blood metabolites, hormones, and rumen fermentation characteristics were analyzed. Blood β-hydroxybutyric acid and insulin levels were high, whereas blood amino acid levels were low in cattle with high methane emissions. Further, propionate and butyrate levels differed depending on the enteric methane emissions. Hepatic genes, such as *SERPINI2, SLC7A5, ATP6*, and *RRAD*, which were related to amino acid transport and glucose metabolism, were upregulated or downregulated during the late fattening phase. The above mentioned metabolites and liver transcriptomes could be used to evaluate enteric methanogenesis in Japanese Black cattle.

## Introduction

Enteric methane emitted by domestic animals is of primary concern in the livestock industry. Ruminants generally have higher methane emissions than monogastric animals because of a long digestive tract and an extensive ecosystem in the rumen that is inhabited by numerous microorganisms, including methanogens. Methane is the final product of ruminal fermentation. Feedstuff ingested by ruminants is broken into monomer nutrients by ruminal microorganisms. The nutrients are then fermented to volatile fatty acids (VFAs), such as acetate, propionate, and butyrate. These VFAs are absorbed along the ruminal epithelium and utilized as energy for cattle growth and production. During these metabolic processes, a large amount of metabolic H_2_ is generated as a by-product of fermentation. Subsequently, the methanogens use the generated H_2_ to promote the reduction of CO_2_ to produce methane, which is consequently, emitted outside the body. In ruminants, methanogenesis consumes 2–15% of the total energy intake^1^. To compensate for this energy loss, more feedstuffs are required; additionally, greenhouse gases are generated while producing feed resources that eventually accelerate global warming. From the producer's perspective, the energy loss in livestock due to methane production increases the production costs and decreases the economic efficiency.

The dietary strategies to reduce methane emissions from cattle focus on changing the feedstuff composition^2,3^ or using chemical^4^ and natural additives^5–9^. The additives are mainly developed to inhibit rumen microbial activity and feed is modified to change the composition of the fermentation products in the rumen. Current studies on methane emissions are limited to the rumen ecosystem, and do not sufficiently explain the complex mechanisms involved in methanogenesis. In addition, the metabolic effects of other organs, such as the liver, have not been considered. Theoretically, reducing methane emissions is thought to lead to an increase in the energy used for livestock growth and production; however, reducing methane emissions does not necessarily lead to improvements in production. To reveal these unexplained results, investigating the overall physiological changes related to methane production in cattle is necessary.

In ruminants, the liver regulates energy balance by synthesizing glucose using propionic acid absorbed from the rumen^10^; moreover, it regulates fat metabolism through fat oxidation and synthesis^11^. The liver is responsible for the overall metabolic physiology and for controlling the energy balance. Additionally, the metabolic and pathological conditions of the liver are closely related to livestock productivity. Higgins et al.^12^ reported that the difference in the residual feed intake, a criterion for determining livestock productivity, may result from differences in the physiological processes in the liver. Further, Lancaster et al.^13^ found that differences in feed efficiency of individual cattle are closely related to proteins secreted abundantly from the mitochondria in the liver and muscles. Several studies have reported that methane emissions are low in cattle having low residual feed intake^14,15^. Our previous study on Japanese Black cattle reported that several hepatic genes related to energy metabolism are regulated during the late fattening phase when energy intake is the highest^16^; furthermore, our results suggested that liver metabolism adapts to store excess energy during periods of high energy intake. As liver metabolism is closely related to feed efficiency and energy level, it is believed to directly and indirectly participate during enteric methane production. Additionally, the physiological features of Japanese Black cattle are likely to differ from those of other beef cattle because of different rearing environments and feeding management practices. Therefore, investigating the methane emissions of Japanese Black cattle raised according to the feeding management strategies developed based on the general livestock practices in Japan is necessary. This study aimed to investigate the physiological characteristics associated with enteric methane emissions during the fattening phase in Japanese Black steers (high- and low-emission cattle) bred and raised in Japan. The study provides novel insights to effectively reduce enteric methane emissions based on extensive data involving physiological features, such as blood metabolites and fermented products, and liver transcriptome information of Japanese Black cattle.

## Results

### Blood metabolome and rumen fermentation profiles

**Figure 1:**
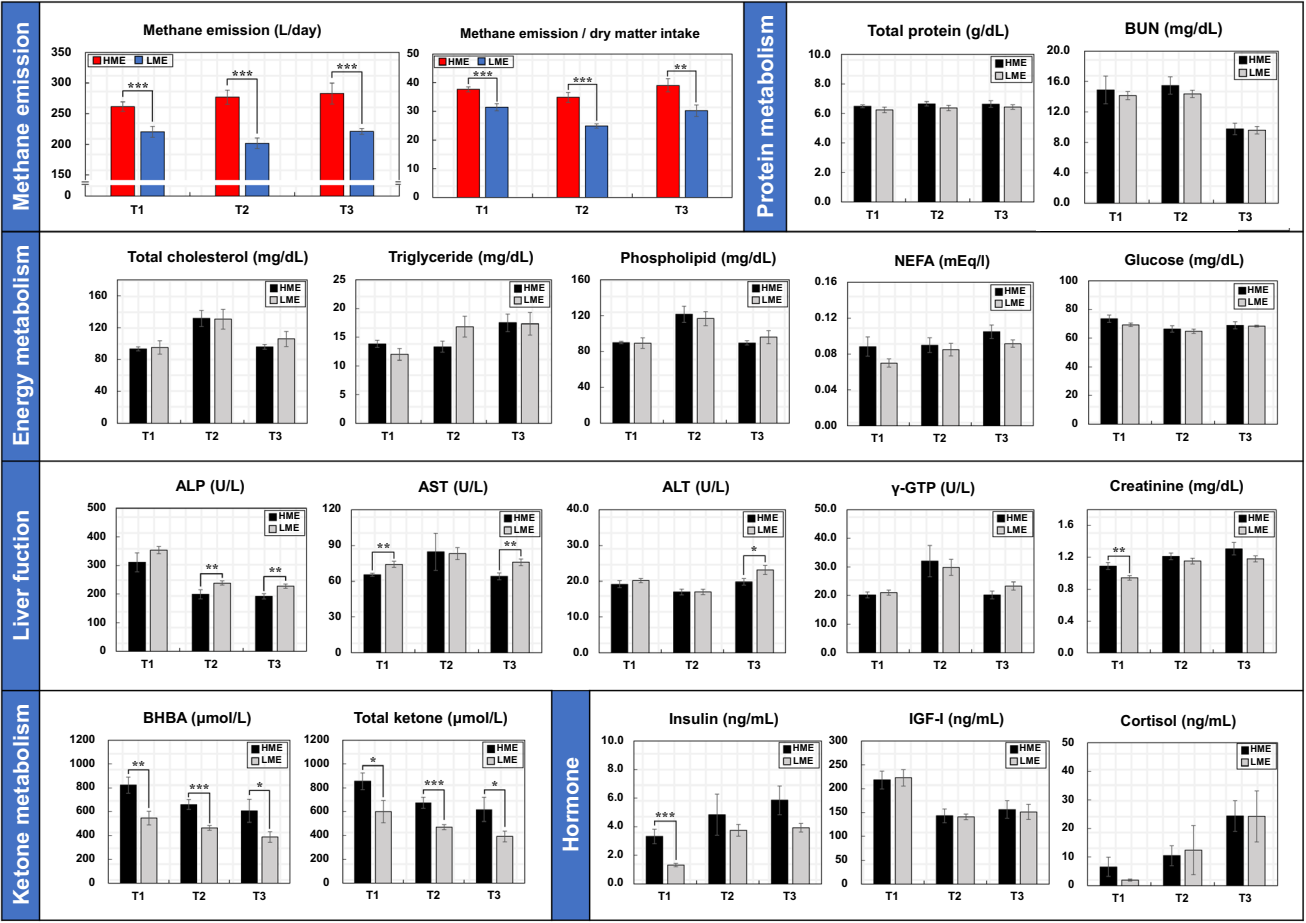
Comparisons of methane emissions and blood metabolite levels in HME vs LME cattle during the fattening period. T1: early fattening phases (13 months of age), T2: middle fattening phases (20 months of age), T3: late fattening phases (28 months of age). HME: group of high methane emission cattle (n = 6), LME: group of low methane emission cattle (n = 6). BUN: blood urea nitrogen, NEFA: non-esterified fatty acid, ALP: alkaline phosphatase, AST: aspartate aminotransferase, ALT: alanine aminotransferase, γ-GTP: gamma(γ)-glutamyl transferase, BHBA: β-hydroxybutyric acid, IGF-I: insulin-like growth factor 1. Data are presented as mean ± SEM. *p < 0.1, **p < 0.05, and ***p < 0.01.

**Figure 2:**
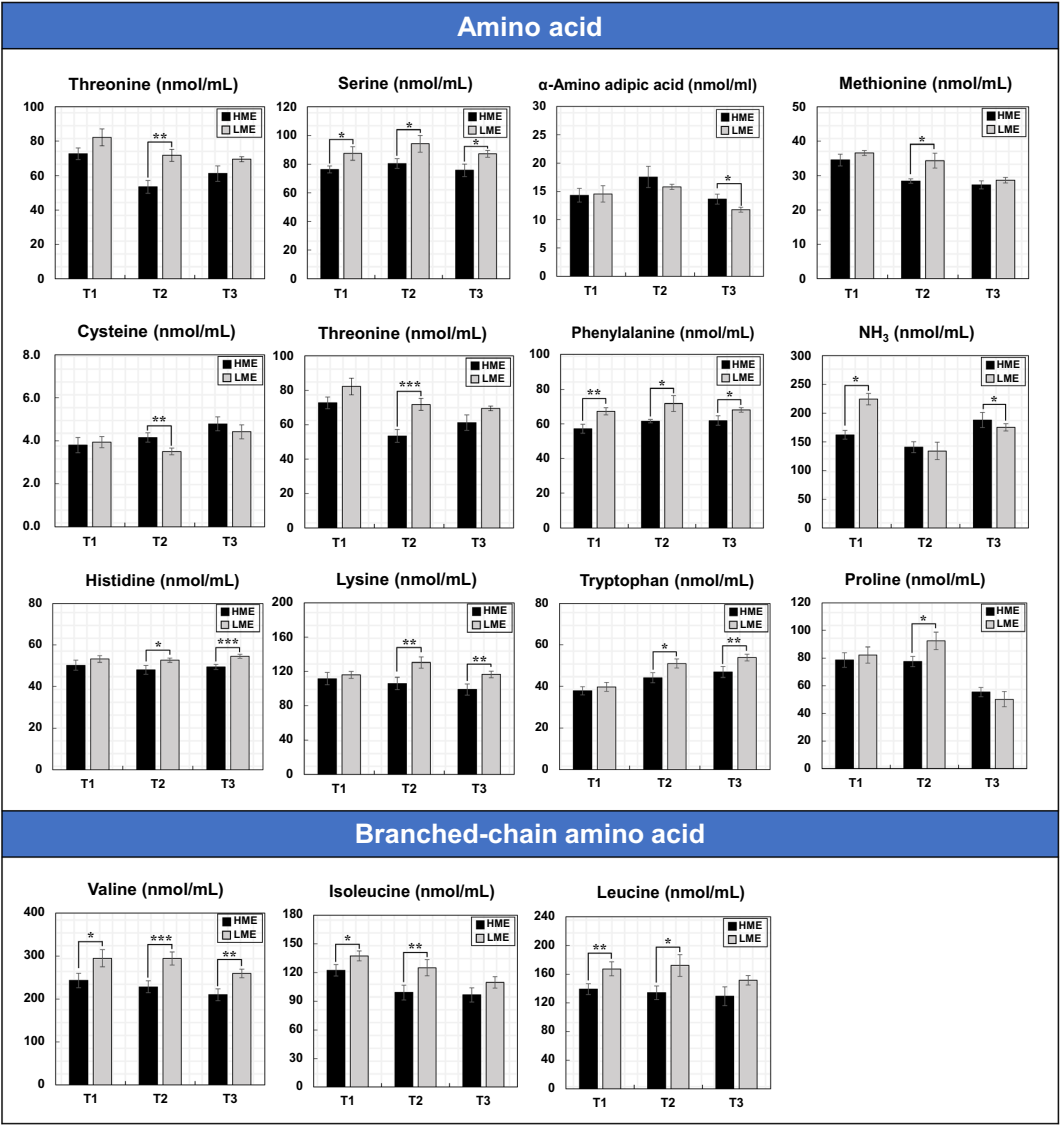
Comparisons of blood amino acid levels in HME vs LME cattle during the fattening period. T1: early fattening phases (13 months of age), T2: middle fattening phases (20 months of age), T3: late fattening phases (28 months of age). HME: group of high methane emission cattle (n = 6), LME: group of low methane emission cattle (n = 6). Data are presented as mean ± SEM. *p < 0.1, **p < 0.05, and ***p < 0.01.

**Figure 3:**
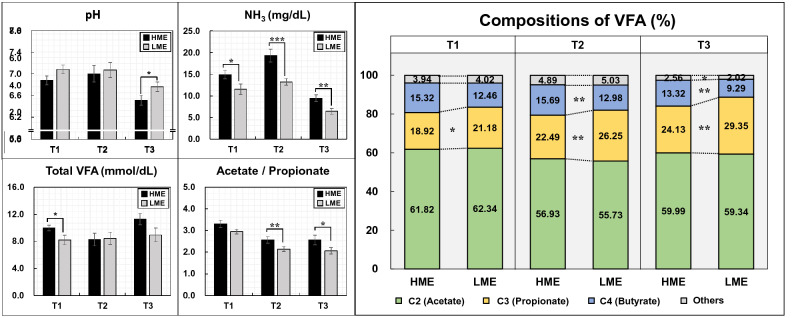
Compositions of rumen fermentations in HME vs LME cattle during the fattening period. T1: early fattening phases (13 months of age), T2: middle fattening phases (20 months of age), T3: late fattening phases (28 months of age). HME: group of high methane emission cattle (n = 6), LME: group of low methane emission cattle (n = 6). VFA: volatile fatty acid. Data are presented as mean ± SEM. *p < 0.1, **p < 0.05, and ***p < 0.01.

Figure 1 shows the methane emissions and blood metabolites levels determined in the high methane emission (HME) and low methane emission (LME) cattle groups during the early (T1), middle (T2), and late (T3) fattening phases. The concentrations of alkaline phosphatase (ALP) and aspartate aminotransferase (AST) significantly (*P* = 0.01) increased in the LME group during T3. However, the concentrations of β-hydroxybutyric acid (BHBA) significantly increased in the HME group during T1 (*P* = 0.02) and T2 (*P* < 0.01). Blood levels of insulin were significantly (*P* < 0.01) higher in the HME group than in the LME group in T1, but the cortisol and insulin-like growth factor 1 (IGF-I) did not show difference during any fattening phases. Figure 2 shows the levels of blood amino acids in the HME and LME groups during fattening period. The concentrations of several blood amino acids, including threonine, valine, histidine, lysine, and tryptophan, increased significantly (*P* < 0.05) in the LME group during T2 and T3, whereas only cysteine levels were significantly (*P* = 0.03) high in the HME group during T2. In the rumen fermentation samples (Fig. 3), the NH_3_ concentration was higher in the HME group during all fattening phases. Moreover, total VFA content increased in the HME group during T1 and T3, but the difference was not significant. The proportion of propionate (%) decreased in the HME group during all fattening phases, whereas butyrate was significantly higher in the HME group during T2 (*P* = 0.02) and T3 (*P* = 0.03). Supplementary Tables S1–S3 online lists the concentrations of blood metabolome and rumen fermentation during fattening period.

### Growth performances, carcass traits, and methane emissions

Tables 1 and 2 summarize the growth performances, feed intake, and carcass traits of both HME and LME Japanese Black cattle. The average body weight at the slaughter was slightly higher in the HME group, but the difference in the daily gain and feed intake was not significant. Carcass weights in the HME and LME groups were 478.33 kg and 468.17 kg, respectively, and eye muscle areas were 59.33 cm^2^ and 56.33 cm^2^, respectively. Although the carcass traits related to meat quantity were slightly lower in the LME group than in the HME group, the difference was not significant. Moreover, no significant difference was observed in the beef marbling score (BMS) between the two groups. The average methane emissions (L/day) for the HME and LME groups were 261.57 L/day and 220.40 L/day, 276.94 L/day and 201.67 L/day, and 282.89 L/day and 221.15 L/day during T1, T2, and T3, respectively (Fig. 1; Supplementary Table S1).

**Table 1:** Mean growth performance and feed intake in Japanese black cattle during the fattening period.

Variable	T1		T2		T3	
	HME	LME	HME	LME	HME	LME
**Growth performance**						
Average body weight (kg)	398.33 (385.50–407.50)	387.50 (372.00–405.00)	590.83 (567.00–643.57)	562.50 (525.86–600.57)	730.48 (675.71–788.86)	724.48 (676.86–777.14)
Average daily gain (kg/day)	0.78 (0.45–1.07)	0.89 (0.84–0.93)	0.88 (0.70–1.04)	0.83 (0.69–0.94)	0.61 (0.52–0.71)	0.66 (0.59–0.80)
**Feed intake (kg/day)**						
Concentrate	5.23 (5.06–5.33)	5.32 (5.30–5.33)	7.07 (6.02–8.29)	7.19 (6.24–8.51)	6.44 (6.02–7.55)	6.64 (5.24–7.83)
Rice straw	1.27 (0.85–1.70)	1.39 (0.85–1.70)	0.70 (0.51–0.95)	0.80 (0.51–1.02)	0.61 (0.43–0.79)	0.68 (0.43–0.79)
Kraft pulp feed	0.44 (0.00–0.85)	0.29 (0.00–0.85)	0.23 (0.00–0.51)	0.15 (0.00–0.46)	0.22 (0.00–0.44)	0.14 (0.00–0.37)
Dry matter	6.94 (6.76–7.04)	7.01 (6.94–7.04)	8.01 (6.86–9.04)	8.14 (7.09–9.52)	7.26 (6.67–8.21)	7.45 (6.03–8.12)
Total digestible nutrients	5.23 (4.99–5.50)	5.23 (5.06–5.48)	6.40 (5.55–7.22)	6.47 (5.56–7.52)	5.84 (5.32–6.59)	5.97 (5.52–7.05)
Crude protein	0.89 (0.87–0.93)	0.91 (0.89–0.93)	1.05 (0.87–1.23)	1.07 (0.94–1.27)	0.80 (0.72–0.94)	0.83 (0.65 -0.97)

T1: early fattening phase (13 months of age), T2: middle fattening phases (20 months of age), T3: late fattening phases (28 months of age). HME: group of high methane emission cattle (n = 6), LME: group of low methane emission cattle (n = 6); The cattle were fed concentrates as follows: T1: steam-flaked corn 42%, wheat bran 27%, corn gluten meal 10%, soybean meal 12%, soybean hull 6%, salt 1% (TDN 71.2%, CP 15.9%); T2: steam-flacked corn 42%, barley 14%, wheat bran 21%, corn gluten meal 5%, soybean meal 10%, soybean hull 6%, salt 1% (TDN 72.5%, CP 14.4%); T3: steam-flacked corn 44%, barley 25%, wheat bran 14%, soybean meal 5%, soybean hull 10%, salt 1% (TDN 72.8%, CP 12.0%). The rice straw intakes were as follows: DM 87.8%, TDN 37.7%, CP 4.7%. The kraft pulp feed intakes were as follows: DM 76.9%, TDN 51.1%, CP 0.3%.

**Table 2:** Mean carcass traits in HME vs LME.

Variable	HME	LME	SEM	*P* value
Live weight (kg)	779.67	760.00	10.17	0.31
Growth rate (kg/day)	0.84	0.82	0.01	0.49
Carcass weight (kg)	478.33	468.17	7.84	0.49
Eye muscle area (cm^2^)	59.33	56.33	1.83	0.39
Rib thickness (cm)	8.15	7.75	0.19	0.31
Subcutaneous fat (cm)	3.02	2.80	0.21	0.59
BMS	8.33	8.33	0.43	0.94

HME: group of high methane emission cattle (n = 6), LME: group of low methane emission cattle (n = 6). Values indicate mean. SEM: standard error of the mean, BMS: beef marbling score (Japanese standards ranged from 1, which contains no visible marbling, up to 12, which is heavily marbled).

### Gene expression analysis by RNA-Seq

In total, 1,609 million reads and 162,518 million base pairs were generated from RNA sequencing of liver tissue during the entire experimental period. The average number of reads acquired from the HME group were 47.6, 48.3, and 52.9 million in T1 (n = 5), T2 (n = 6), and T3 (n = 5), respectively, and the clean read rates were 95.0%, 95.9%, and 95.9% in the respective phases (Supplementary Tables S5–S6). Moreover, the average number of reads acquired from the LME group were 50.3, 53.0, and 49.9 million in T1 (n = 6), T2 (n = 5), and T3 (n = 5), respectively, and the corresponding clean read rates were 95.4%, 95.8%, and 95.8% in each phase, respectively (Supplementary Tables S5– S6). On average, 15,411, 15,977, and 16,116 expressed genes were detected in the liver tissue of the HME cattle in T1, T2, and T3, respectively, whereas 15,810, 15,933, and 16,085 expressed genes were detected in the liver tissue of the LME cattle in T1, T2, and T3, respectively (Supplementary Tables S5–S6).

**Figure 4:**
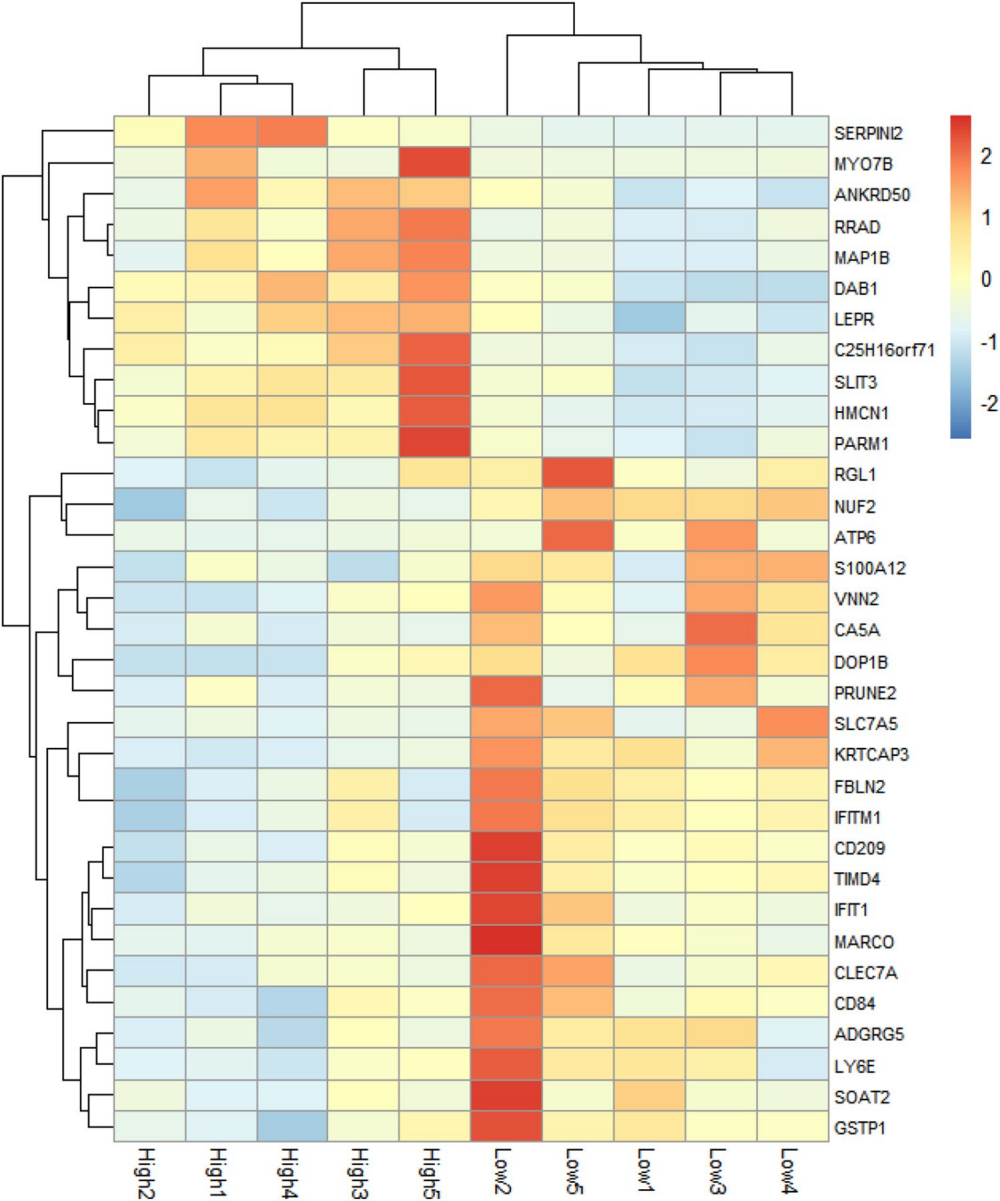
Heat map illustrating the hierarchical cluster of differentially expressed genes in HME vs LME in late fattening phases. HME: group of high methane emission cattle (n = 5), LME: group of low methane emission cattle (n = 5).

To determine differentially expressed genes (DEGs) in the liver tissues of both HME and LME groups in each phase (T1, T2, and T3), we defined hepatic genes as DEGs according to the following criteria: a > 1.0 log_2_ fold change in expression level, base mean > 50, and false discovery rate (FDR) < 0.05. In total, 56 DEGs were identified between the HME and LME groups, with 1 (downregulated), 11 (6 upregulated and 5 downregulated), and 44 (14 upregulated and 30 downregulated) DEGs found in T1, T2, and T3, respectively (Supplementary Table S7). Figure 4 shows a heat map illustrating the hierarchical cluster of DEGs of the HME and LME groups in T3. Cluster hierarchization using DEGs confirmed the presence of two distinct groups. Table 3 lists the top 10 DEGs in the order of the greatest expression difference between the groups. The serpin family I member 2 (*SERPINI2*) with a log_2_fold value of 5.01, showed up-regulated expression in the HME group. The down-regulated gene, solute carrier family 7 member 5 (*SLC7A5*), showed the greatest expression difference between the HME and LME groups.

**Table 3:** Top 10 differentially expressed genes in HME vs LME in late fattening phases.

Gene name	Description	*P* value	log_2_FoldChange
*SERPINI2*	Serpin family I member 2	< 0.01	5.01
*MYO7B*	Myosin VIIB	< 0.01	4.61
*SLC7A5*	Solute carrier family 7 member 5	< 0.01	− 2.63
*HMCN1*	Hemicentin 1	< 0.01	2.12
*KRTCAP3*	Keratinocyte associated protein 3	< 0.01	− 2.08
*PRUNE2*	Prune homolog 2 with BCH domain	< 0.01	− 1.81
*MAP1B*	Microtubule associated protein 1B	< 0.01	1.69
*SLIT3*	Slit guidance ligand 3	< 0.01	1.57
*ATP6*	Mitochondrially encoded ATP synthase membrane subunit 6	< 0.01	− 1.54
*RRAD*	Ras related glycolysis Inhibitor and calcium channel regulator	< 0.01	1.53

DEGs: differentially expressed genes.

### Gene ontology analysis of DEGs

Gene ontology (GO) analysis was conducted by running queries for each DEG against the GO database, which provided data on biological processes (BP), cellular components (CC), and molecular functions (MF) associated with the DEGs. GO analysis of the DEGs in the HME and LME groups during T3 showed the highest difference in gene expression. The DEGs during T3 were mainly involved in immune and xenobiotic responses (Tables 4,5; Supplementary Figure S1), such as leukocyte activation involved in immune response (GO: 0002366), cell activation involved in immune response (GO: 0002263), xenobiotic metabolic process (GO: 0006805), cellular response to xenobiotic stimulus (GO: 0071466), mast cell degranulation (GO: 0043303), response to fungus (GO: 0009620), mast cell-mediated immunity (GO: 0002448), response to xenobiotic stimulus (GO: 0009410), and mast cell activation involved in the immune response (GO: 0002279). The CC- and MF-related genes were involved (Table 4) in extracellular interaction and substance transport processes, including extracellular region (GO: 0005576), extracellular exosome (GO: 0070062), calcium ion binding (GO: 0005509), and sulfur compound binding (GO: 1901681).

**Table 4:** Gene ontology of differentially expressed genes in HME vs LME in late fattening phases.

Ontology	GO Term ID	Description	No. of DEGs	P-value
Biological processes	GO:0016192	vesicle-mediated transport	6	0.01
	GO:0051640	Organelle localization	4	0.02
	GO:0045321	Leukocyte activation	4	0.06
	GO:0001775	Cell activation	4	0.08
	GO:0006909	Phagocytosis	3	0.01
	GO:0050770	Regulation of axonogenesis	3	0.02
	GO:0002366	Leukocyte activation involved in immune response	3	0.04
	GO:0002263	Cell activation involved in immune response	3	0.04
	GO:0010769	Regulation of cell morphogenesis involved in differentiation	3	0.06
	GO:0010975	Regulation of neuron projection development	3	0.07
	GO:0051656	Establishment of organelle localization	3	0.07
	GO:0007409	Axonogenesis	3	0.09
	GO:0061564	Axon development	3	0.10
	GO:0006805	Xenobiotic metabolic process	2	0.03
	GO:0071466	Cellular response to xenobiotic stimulus	2	0.04
	GO:0043303	Mast cell degranulation	2	0.04
	GO:0009620	Response to fungus	2	0.04
	GO:0002448	Mast cell mediated immunity	2	0.04
	GO:0009410	Response to xenobiotic stimulus	2	0.04
	GO:0002279	Mast cell activation involved in immune response	2	0.05
	GO:0032418	Lysosome localization	2	0.06
	GO:0045576	Mast cell activation	2	0.06
	GO:0043299	Leukocyte degranulation	2	0.07
	GO:0002275	Myeloid cell activation involved in immune response	2	0.08
	GO:0002444	Myeloid leukocyte mediated immunity	2	0.09
Cellular composition	GO:0005576	Extracellular region	10	0.04
	GO:0044421	Extracellular region part	9	0.05
	GO:0070062	Extracellular exosome	7	0.09
	GO:1903561	Extracellular vesicle	7	0.09
	GO:0043230	Extracellular organelle	7	0.09
	GO:0031012	Extracellular matrix	3	0.10
	GO:0005903	Brush border	2	0.10
Molecular functions	GO:0005509	Calcium ion binding	4	0.05
	GO:1901681	Sulfur compound binding	3	0.02

GO: gene ontology, DEGs: differentially expressed genes.

**Table 5:** Gene ontology of differentially expressed genes related to immune activity in HME vs LME in late fattening phases.

GO Term ID	Description	No. of DEGs	*P* value	DEGs
GO:0002366	Leukocyte activation involved in immune response	3	0.04	*CD84, CLEC7A, S100A12*
GO:0002263	Cell activation involved in immune response	3	0.04	*CD84, CLEC7A, S100A12*
GO:0006805	Xenobiotic metabolic process	2	0.03	*GSTP1, S100A12*
GO:0071466	Cellular response to xenobiotic stimulus	2	0.04	*GSTP1, S100A12*
GO:0043303	Mast cell degranulation	2	0.04	*CD84, S100A12*
GO:0009620	Response to fungus	2	0.04	*CLEC7A, S100A12*
GO:0002448	Mast cell mediated immunity	2	0.04	*CD84, S100A12*
GO:0009410	Response to xenobiotic stimulus	2	0.04	*GSTP1, S100A12*
GO:0002279	Mast cell activation involved in immune response	2	0.05	*CD84, S100A12*

GO: gene ontology, DEGs: differentially expressed genes.

## Discussion

**Figure 5:**
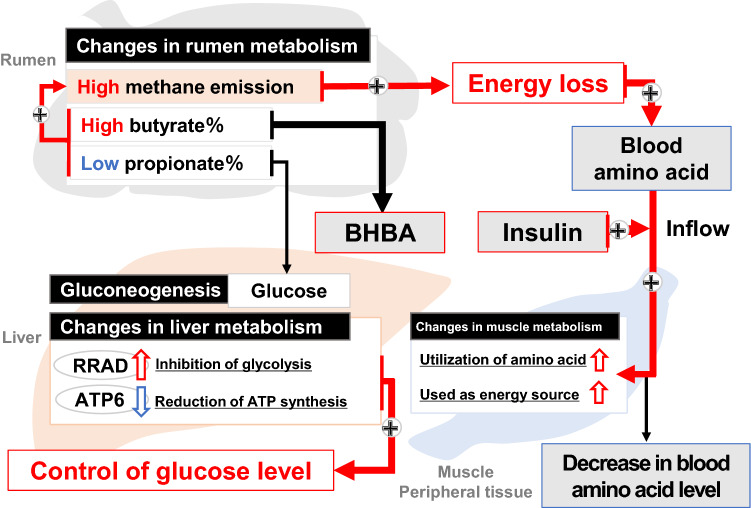
Proposed model for physiological parameters and hepatic transcriptomes related with high methane emission in Japanese Black cattle. The cattle with high methane emission seems to actively utilize the amino acid to replenish for the loss of energy used in methane production, resulting in a decrease in blood amino acid levels and an increase in blood insulin concentration with no changes of growth and productivity.

Based on the enteric methane emissions, we comprehensively investigated the physiological status of Japanese Black steers in terms of various parameters, such as liver transcriptome, blood metabolites, hormones, and rumen fermentation characteristics. The major physiological changes associated with the methane emission levels have been discussed in the subsequent section (Fig. 5).

### Changes in the physiological parameters according to the enteric methane emissions

This section describes the changes in metabolite profiles, hormones, and rumen fermentation characteristics according to the enteric methane emissions from Japanese Black steers. The BHBA concentration and the levels of total ketone bodies were higher in the HME cattle than in the LME cattle during all fattening phases. Generally, ketone bodies, such as BHBA increase under the negative energy balance condition and are produced as metabolites during the lipid oxidation of fatty acids in the liver. Further, the butyrate produced in the rumen is absorbed through the rumen epithelium, and the absorbed butyrate is converted to BHBA^17^, which is then transported through the bloodstream and utilized as energy in various tissues. Thus, in this study, the high BHBA concentrations observed in the serum of the HME cattle may be partially due to the high rate of butyrate production in the rumen.

Regarding the rumen fermentation compositions, the HME group showed a higher butyrate ratio and lower propionate ratio than the LME group; correspondingly, the ratio of acetate and propionate was high. Bénédict et al.^18^ reported that cattle, which showed low methane emissions because of anti-methanogenic compounds, showed a higher propionate ratio and lower acetate ratio than the control group with relatively high methane emissions. Supplementation of disodium fumarate decreased the ratio of acetate and propionate, thereby decreasing methane production. In addition, the methanogen levels were significantly different between the high and low methane emission groups depending on the proportion of fumarate added and of the roughage and concentrate diets^9^. Another study reported that cattle fed with chloroform, which is effective in reducing methane emissions, have similar features, that is, relatively lower acetate and higher proportionate ratios^19^. In summary, previous studies and the findings of our study indicated that the production of ruminal propionate decreased in the HME cattle compared to the LME cattle. Despite the differences in the cattle breed and dietary conditions, this tendency indicated a close relationship between methane emissions and propionate concentration. However, the relationship between butyrate and methane emissions has not yet been clearly identified. Based on an in vitro study, Eslam et al.^20^ reported that supplementation with plant bioactive extracts reduced enteric methane and increased both ruminal propionate and butyrate concentrations. Another in vitro experiment reported that the methane reduction effect of resveratrol was affected by the rate of forage and concentrate, and the ruminal butyrate levels in resveratrol treatment differed according to dietary differences^21^. Further, the relationship between ruminal butyrate and methane emissions was greatly influenced by the cattle breed, amount of feed intake, and management methods; thus, the increased butyrate levels in the HME cattle were believed to reflect the typical physiological characteristics of Japanese Black cattle. Moreover, the changes in rumen fermentation were believed to be due to differences in the rumen microbial communities. In this study, the dietary composition and feed intake did not differ between the HME and LME groups; therefore, the differences in the rumen fermentation could be attributed to the rumen microbial communities, which originally inhabited the rumen, rather than dietary changes. In addition, ruminal production of acetate and butyrate promotes methanogenesis by releasing dihydrogen, whereas ruminal production of propionate reduces methanogenesis rate by consuming dihydrogen^18^, thus, suggesting that the composition of ruminal VFAs changes according to the presence of ruminal microorganisms, and the ruminal products in turn affect methanogenesis.

Approximately 80–85% of the ruminal propionate absorbed through the portal vein in ruminants is used as the main substrate for gluconeogenesis in the liver, while the remaining 20% is transported to each tissue through blood vessels^22,23^. In the present study, as the ruminal production of propionate leads to glucose production through gluconeogenesis in the liver, glucose production might be lower in the HME cattle, which had a low propionate ratio. However, blood glucose concentrations did not differ between the two groups. Therefore, these findings suggested that the HME cattle maintain glucose production by regulating gluconeogenesis to compensate for the relatively low level of ruminal propionate and increased energy loss owing to methane production.

Insulin stimulates protein metabolism directly by activating the translational components^24,25^. In particular, branched-chain amino acid (BCAA), such as valine, leucine, and isoleucine is less metabolized in the liver and are mainly used as energy sources in muscle and peripheral tissues^26^. The BCAA inflow into muscle tissues is catalyzed by insulin signals. Ammonia produced by BCAA metabolism in muscles and peripheral tissues is converted into alanine by the transition reaction of amino groups (-NH_2_); moreover, the alanine is transported to other organs, such as the liver and kidney, for disposal^27^. Mackle et al.^28^ reported that the upregulation of blood insulin levels using the hyperinsulinemic euglycemic clamp technique reduced the circulating levels of amino acids by 33%, especially BCAA by 41% in lactating cows. Therefore, these findings revealed that the HME cattle actively used amino acids as energy sources in muscle and other peripheral tissues to supplement the energy loss associated with methane production, resulting in decreased proportion of amino acids. Insulin possibly stimulated amino acid transport, which may possibly explain why insulin was maintained at a high level in the HME cattle. Bénédict et al.^18^ investigated the changes in the concentration of blood metabolites in Holstein cows supplemented with anti-methanogenic compounds and reported that the blood amino acid concentration was relatively lower in the high methane cattle than in the low methane cattle. Moreover, they suggested that the changes in the ruminant microbial communities due to anti-methanogenic compounds might have changed the production ratio of rumen fermentations, including amino acids. Another study reported that the concentrations of glycine and methyl-histidine significantly decreased in the low methane group supplemented with fumarate, but there was no difference in the concentration of other amino acids, including BCAA^29^. Accordingly, the previous and present study suggested that the relationship between methane emissions and blood amino acid levels differed depending on the cattle breed and diet, and thus, further investigations are warranted.

### Liver transcriptome changes according to the enteric methane emissions

We conducted GO analysis for enriched DEGs to define the functional roles of DEGs depending on the enteric methane emissions. We focused on the comparisons between the HME and LME groups in the late fattening phase, which showed the greatest differences in gene expression in the DEG analysis. Further, the DEGs were significantly enriched in vesicle-mediated transport and immune activity. The expression of genes, such as *SERPINI2, SLC7A5,* mitochondrially encoded ATP synthase membrane subunit 6 (*ATP6*)*,* and Ras related glycolysis inhibitor and calcium channel regulator (*RRAD*) differed in the HME and LME groups during the late fattening phase. Although the physiological roles of *SERPINI2* have not yet been described thoroughly, this gene is important to regulate various metabolic processes and maintain cellular function as a protease inhibitor, similar to other serpins (serine protease inhibitors)^30^. Lotus et al.^31^ reported that defects in *SERPINI2* caused apoptosis in pancreatic acinar cells, resulting in functional defects in immune cells and tissue integrity. In addition, several studies^32–35^ reported that SERPINI2 is positively correlated to feed conversion ratio (FCR) and residual feed intake (RFI), an indicator of feed efficiency in livestock. Regarding the relationship between RFI and SERPINI2, Chen et al.,^34^ mentioned that SERPINI2, which is involved in various cellular processes, influences the nutritional metabolism and growth of animals to some extent, which could be used as an indicator of feed efficiency in cattle feeding management. In the present study, the FCR was slightly higher (unpublished data; HME 9.57 vs. LME 7.19, *P* = 0.2) in the HME group during T1. Although the FCR data did not show a large difference between the HME and LME groups similar to that observed in previous studies, the marginal difference in the FCR may have affected the expression of *SERPINI2*. *SLC7A5*, which is a sodium-independent high-affinity amino acid transporter, mediates the cellular uptake of large neutral amino acids, such as phenylalanine, tyrosine, leucine, and tryptophan, and promotes protein synthesis, for cell growth and proliferation by activating the *mTORC1* signaling pathway^36–38^. We hypothesized that the liver and internal metabolism in Japanese Black cattle changed to actively use other energy sources, such as amino acids, to supplement energy loss caused by methane production. Consequently, there was no difference in the feed intake, glucose concentration, and growth characteristics between the HME and LME groups. However, marginal changes in *SLC7A5*, an amino acid transporter, and in the gene expression associated with the transportation and glycosylation of amino acids in the liver, increased in the LME group. Therefore, our findings implied the following: First, liver *SLC7A5* expression increased due to the relatively high concentration of blood amino acids in the LME group. Previous studies^39^ reported that blood amino acid concentrations increased with increasing essential amino acid intake, and that *SLC7A5* gene expression in muscle tissue also increased. Second, SLC7A5 may have mediated protein metabolism more sensitively in other tissues, such as muscle tissues, than the liver. BCAA is mainly metabolized in muscle tissues, and SLC7A5 induces the BCAA metabolic process^37^. Based on our findings, which reported a significant difference in the blood BCAA concentration in the blood amino acids between the HME and LME groups, the HME cattle seemed to actively utilize the BCAA in the muscle and other peripheral tissues, except the liver, to maintain energy metabolism. However, in this study, the expression of intramuscular *SLC7A5* gene expression was not assessed, and thus, further investigations should be conducted.

*ATP6*, which is involved in the mitochondrial ATPase activity, is necessary for the final step of oxidative phosphorylation in the electron transport chain for ATP synthesis, and plays a critical role in energy metabolism^40,41^. *RRAD*, which is regulated by *p53*, regulates aerobic glycolysis^42^. In addition, *RRAD* inhibits the translocation of GLUT1 to the plasma membrane^42^. Previous studies reported^43,44^ that the overexpression of *RRAD* inhibits insulin-stimulated glucose uptake in mouse muscle cells and adipocytes. These findings suggested that the mechanisms responsible for inhibiting glucose uptake by RRAD are still unclear; nevertheless, RRAD may have played a role in providing a link between insulin resistance, hyperinsulinemia, and atherosclerosis. In this study, *ATP6* was downregulated and *RRAD* was upregulated in the HME group; moreover, both genes were related to energy metabolism. As mentioned previously, the ruminal production ratio of propionate was relatively low in the HME cattle, which would have decreased liver gluconeogenesis. Therefore, suppressing glycolysis and preventing the consumption of glucose, which serves as an energy source, in the liver is necessary to maintain sufficient glucose levels during energy metabolism. The present study suggested that ATP6 and *RRAD* genes likely participated in maintaining blood glucose levels in cattle.

Accordingly, the expression of genes, such as S100 calcium-binding protein A12 (*S100A12*) and C-type lectin domain family 7 member A (*CLEC7A*), which are related to immune activity, was decreased in the HME group during T3. *S100A12* belongs to the S100 protein family, the largest subgroup of EF-hand calcium-binding proteins, and is predominantly expressed and secreted in neutrophil granulocytes. It plays a role in developing an immune response induced by microorganisms and parasites and is also involved in autoimmune responses^45,46^. S100A12 activates the pro-inflammatory reaction by interacting with the corresponding receptor for advanced glycation end products (RAGE)^47,48^, leading to cytokine production, chemotaxis, and increased oxidative stress^45,49^. Therefore, S100A12 is used as a subclinical indicator of inflammation or microbial infection, and the increase in the blood concentration of S100A12 protein has been associated with Type 2 diabetes, atherosclerosis-related inflammation, and tumorigenic processes^50–52^. A previous study reported an increase in the protein levels associated with the interaction between S100A12 and RAGE in Type 2 diabetes patients compared to normal controls, possibly due to increased oxidative stress caused by persistent hyperglycemic conditions^50,51^. Another study reported that the blood concentrations of S100A12 and RAGE decreased based on the insulin treatment, maybe because of the hypoglycemic action of insulin^53^. In this study, the difference in *S100A12* gene expression between the HME and LME groups was probably because of factors other than oxidative stress, which was caused by hyperglycemia. This is because insulin concentration differed, but blood glucose levels did not differ, with all concentrations being within the normal range.

*CLEC7A* is a member of the C-type lectin/C-type lectin-like domain (CTL/CTLD) superfamily and is predominantly secreted in myeloid dendritic cells, monocytes, macrophages and B cells^54^, and in the lungs and liver. CLEC7A functions as a pattern-recognition receptor for various glucans derived from fungi and plants, and plays an important role in generating innate immune responses^55^. In general, the ruminal ecosystem inhibits unsuitable immune responses by recognizing the symbiotic bacteria in the mucus layer and activating the tolerance signals; however, bacteria introduced into the bloodstream sometimes affect other organs, thereby causing severe immune responses or pathological problems. Ruminal bacteria, such as *Fusobacterium necrophorum* and *Arcanobacterium pyogenes*, the proportion of which increases during acidosis, are transferred to the liver through the bloodstream, causing liver abscess^56,57^. In this study, the upregulated *CLEC7A* gene in the LME group may have been due to the activation of the immune response caused by bacterial influx into the liver.

## Conclusion

This study showed that Japanese Black cattle have different physiological characteristics based on the enteric methane emissions. Cattle exhibiting high enteric methane have a higher butyrate and lower propionate ratio in the composition of rumen fermentation parameters; regarding the blood metabolite profiles, the concentration of amino acids decreased, while ketone bodies and insulin increased in HME cattle. Hepatic DEGs related to amino acid and glucose metabolism, such as *SERPINI2* and *RRAD* expression, were upregulated in HME cattle, whereas *SLC7A5* and *ATP6* expression was down-regulated. The cattle with high methane emissions actively utilized amino acids to replenish the energy lost during methane production, thereby decreasing blood amino acid levels and increasing blood insulin concentration without changing the growth and productivity of cattle. Our results suggested that physiological differences and liver transcriptome could be used as parameters to monitor the levels of methane emissions from Japanese Black steers and other similar cattle breeds. Accordingly, the relationship revealed in this study between the various physiological parameters and methane emissions might provide a new perspective different from the existing methane reduction studies.

## Methods

### Animals and experimental design

Animal experiments were performed at the Hyogo Prefectural Technology Center of Agriculture, Forestry and Fisheries (Hyogo Prefecture, Japan) and conducted according to the Guidelines for the Institute of Livestock and Grassland Science^58^ and the Ethical guidance of the Hyogo Prefectural Institute of Agriculture of Forestry and Fisheries Animal Care and Use Committee. The experimental protocol was evaluated and approved by the Hyogo Prefectural Institute of Agriculture of Forestry and Fisheries Animal Care and Use Committee (approval number: H2018-01). All animal experiments were performed in accordance with the ARRIVE guidelines (https://arriveguidelines.org).

Twenty-one Japanese Black steers aged 12 months (initial body weight, 335.6 ± 19.8 kg) were reared until 30 months of age (final body weight, 742.1 ± 49.9 kg). The experimental period was divided into early fattening (12–14 months of age; T1), middle fattening (15–22 months of age; T2), and late fattening phases (23–30 months of age; T3). Experimental animals were fed concentrate and roughage (rice straw and kraft pulp feed) twice daily (at 09:30 and 15:00). Water was always freely available, and other feeding management was conducted in accordance with the practices of the Hyogo Prefectural Technology Center. All steers were fed with specific amounts of formula diet that were changed during each fattening period, as previously reported^16^. After the feeding experiment, the residual methane emissions for each animal were calculated as the difference between the predicted and estimated values of methane emissions (described comprehensively in the next section). Based on the calculated value of residual methane emissions for the entire fattening period, six Japanese Black steers with the highest (HME) and lowest methane emissions (LME) were selected to investigate changes in the liver transcriptome and physiological parameters. The values of residual methane emissions are shown in Supplementary Table S4, and the values of feed intake and growth performance of the HME and LME groups are shown in Table 1 and 2.

### Measuring methane emissions

Methane emissions were measured at the early fattening (T1, 13 months of age), middle fattening (T2, 20 months of age), and late fattening phases (T3, 28 months of age) in 21 Japanese Black steers. Methane concentration was measured for 6 min while feeding the concentrate after roughage feeding. Measurements were repeated six times for three consecutive days in each fattening period. A portable gas collector was placed in each feed tank, and the concentrations of captured CH_4_ and CO_2_ were monitored and recorded using a Micro-Portable Greenhouse Gas Analyzer (Model 909–0050, LGR Inc., CA, USA). The methane emissions of Japanese Black cattle were calculated as follow;

Methane emission (L/day) = (Heat production (HP)/4.9^59^) × CH_4_/CO_2_/100) × 1000 × estimated respiratory quotient^60,61^.

Because the calculated values of methane emissions were measured thrice in T1, T2, and T3 phases, the estimated values of the methane emission were calibrated using a mixed model analysis of variance as follows:

$${\text{Yijk}} = \mu + {\text{Ti}} + {\text{Rij}} + {\text{Pk}} + \left( {{\text{TP}}} \right){\text{ik}} + {\text{eijk}}$$

where Yijk, which represents methane emissions, is a dependent variable, μ is the overall mean, Ti is the fixed effect of treatment i, Rij is the random effect of animal j in Ti, Pk is the fixed effect of fattening period k, (TP)ik is the fixed interaction effect of Ti × Pk, and eijk is the random error. Accordingly, we calculated the predicted values of methane emissions through a linear regression model of dry matter intake to evaluate the methane emission levels. The difference between the estimated value and the predicted value by the linear regression model was considered as residual methane emission (RME). The top six and bottom six individuals of RME were designated as HME (23.35 ± 4.93) and LME (− 19.33 ± 4.03) group, respectively.

### Sample preparation and carcass trait assessment

Experimental samples, including blood, rumen fluid, and liver tissue, were collected at the early fattening (13 months of age), middle fattening (20 months of age), and late fattening phases (28 months of age) in 21 Japanese Black cattle. Twelve samples of blood and rumen fluid were used for profiling analysis in each fattening period, and all physiological parameters were compared between the HME (n = 6) and LME (n = 6) groups. Blood samples were collected at 13:00 h, 3 h after morning feeding from the jugular vein using heparin-sodium tubes (Venoject II VP-H100K; Terumo, Tokyo, Japan). Rumen fluid was collected using a suitable catheter. All samples were adequately treated and stored until metabolic profiling, as described previously^16^. Liver tissue was biopsied from T1; 5 and 6 heads, T2; 6 and 6 heads, and T3; 5 and 6 heads in HME and LME, respectively, according to method described previously^16,62^. All steers were slaughtered at a commercial meat abattoir after a 24 h rest period. Carcasses were chilled for 24 h at 0 °C, after which the left side was opened between the 6th and 7th ribs to evaluate the yield and quality of the carcass according to the standard criteria of the Japan Meat Grading Association^63^. The traits measured during carcass evaluation were carcass weight, beef marbling standard (BMS), rib eye area, rib thickness, and subcutaneous fat thickness.

### Blood metabolome and rumen fermentation profiling

Blood metabolites were analyzed using an automatic biochemical analyzer (Hitachi 7070, Hitachi, Ltd., Tokyo, Japan). The analyses included measurements of total protein, albumin, blood urea nitrogen (BUN), creatinine, total cholesterol, triglycerides, non-esterified fatty acid, glucose, ALP, AST, ALT, lactate dehydrogenase, γ-GTP, creatine kinase, acetate, BHBA, and total ketone bodies. Plasma insulin, IGF-I, and cortisol were assayed by enzyme immunoassay according to the manufacturer’s instructions using a multi-species insulin ELISA kit (Mercodia bovine insulin ELISA, Mercodia AB, Uppsala, Sweden), human IGF-I ELISA kit (Human IGF-I, R&D system, Minneapolis, USA), and cortisol ELISA kit (Cortisol ELISA kit, Enzo Life Sciences Inc., Budapest, Hungary), respectively. After adding trichloroacetic acid to the plasma and filtering the proteins through a membrane filter, the blood amino acid concentrations were determined using a high-speed amino acid spectrometer (L-8900, Hitachi High Tech, Tokyo, Japan). Total VFA and VFA components (acetic acid, propionic acid, butyric acid, and valeric acid) were separated and quantified by gas chromatography (GC2014; Shimadzu, Kyoto, Japan) using a packed glass column (Thermon-3000 [3%]) on a Shimalite TPA 60–80 support (Shinwa Chemical Industries Ltd., Kyoto, Japan). The operating conditions of gas chromatography were as follows: carrier gas, N2; flow volume, 30 mL/min; temperature of the column injection and FID detection, 220 °C; and temperature of the column oven, 140 °C. The ammonium nitrogen concentration in the rumen fluid was quantified by the steam distillation method using an automatic nitrogen analyzer (Kjeltec Auto 1,035; Tecator, Sweden).

### RNA sequencing and data analyses

Total RNA was isolated as described previously^16^. To check whether the purified total RNA could be used in RNA-seq, the RNA integrity number (RIN) was confirmed in a Tape Station 4200 using an RNA Screen Tape kit (AGILENT, Palo Alto, CA, USA), and samples with RIN of higher than 7.0 were used for subsequent experiments. RNA-seq libraries were prepared using the TruSeq stranded mRNA Kit (Illumina) and sequenced using the NovaSeq 6000 platform at Macrogen Japan Corp. The quality of the sequencing reads was evaluated using FastQC software (http://www.bioinformatics.babraham.ac.uk/projects/fastqc, version 0.11.8). Sequencing reads were trimmed using the Trim Galore software (http://www.bioinformatics.babraham.ac.uk/projects/trim_galore/, version 0.5.0) and reassessed using FastQC software. Sequencing reads were mapped to the ARS-UCD1.2 bovine reference genome (ftp://ftp.ensembl.org/pub/release-101/fasta/bos_taurus/dna/) using the HISAT2 software^64^. The String Tie software^64^ was used to calculate the read counts for expressed transcripts with the GTF Bovine gene annotation file (ftp://ftp.ensembl.org/pub/release-101/gtf/bos_taurus). Normalization of counts and PCA were performed using the DESseq2 package^65^ in R statistical software to determine the relationships among individual samples. Two samples from each of T2 and T3 were identified as outliers and removed because they were out of the cluster in the PCA score plot. Thereafter, T1; 5 and 6 samples, T2; 6 and 5 samples, and T3; 5 and 5 samples were used for subsequent data analyses. The DESeq2 package^65^ was also used to detect the differentially expressed genes between HME and LME in each period using normalization of counts and the Wald test. FDR was calculated to account for multiple testing. Significant differentially expressed genes were defined with a > 1.0 log_2_ fold change, base mean > 50, and FDR < 0.05. Functional annotation enrichment analyses using GO^66,67^ were performed using the Database for Annotation, Visualization, and Integrated Discovery (https://david.ncifcrf.gov/summary.jsp). GO terms with *P*-values ≤ 0.05, were considered significantly enriched.

### Statistical analysis

Statistical analyses were performed using SAS software (2012 SAS Institute, Inc., Cary, NC, USA) and R 4.0.3 software (2020 R Foundation for Statistical Computing, Vienna, Austria). All physiological parameters were first assessed for normality and variance homogeneity using the Shapiro–Wilk test and Levene’s test. The significance of the difference between HME and LME in each fattening period was calculated by Student’s t-test and Mann–Whitney test for data meted normal distribution and for those that did not. In the present study, statistical differences were considered significant at *P* < 0.05, and statistical tendencies were indicated by 0.05 < *P* ≤ 0.10.

## Supplementary Information

Supplementary Information 1.

Supplementary Information 2.

Supplementary Information 3.

Supplementary Information 4.

Supplementary Information 5.

Supplementary Information 6.

Supplementary Information 7.

Supplementary Information 8.

Supplementary Information 9.

## Data Availability

RNA sequence data generated from the present study were submitted to the NCBI BioProject database with project number PRJNA760255. The data will be available with the following link: https://www.ncbi.nlm.nih.gov/sra/PRJNA760255.
