# Supplementary material for: Physiological responses and adaptations to high methane production in Japanese Black cattle
Source: Sci Rep. 2022 Jul 1;12:11154. doi: 10.1038/s41598-022-15146-1 (PMC9249741; doi:10.1038/s41598-022-15146-1)
Supplement: Supplementary file 1 — Supplementary Information 1. [file 41598_2022_15146_MOESM1_ESM.docx]

Physiological responses and adaptations to high methane production in Japanese Black cattle

# Minji Kim^1^, Tatsunori Masaki^2^, Kentaro Ikuta^2^, Eiji Iwamoto^2^, Koki Nishihara^1^, Makoto Hirai^1,3^, Yoshinobu Uemoto^1^, Fuminori Terada^1,4^, and Sanggun Roh^1*^

^1^Graduate School of Agricultural Science, Tohoku University, Sendai, 980-8572, Japan

^2^Hyogo Prefectural Technology Center of Agriculture, Forestry and Fisheries, Kasai, Hyogo, 679-0198, Japan

^3^Present address: Central Research Laboratories, Nippon Zenyaku Kogyo Co., Ltd., Koriyama, Fukushima, 963-0196, Japan

^4^Present address: National Institute of Livestock and Grassland Science, National Agriculture and Food Research Organization, Ikenodai, Tsukuba, 305-0901, Japan

*sanggun.roh@tohoku.ac.jp

Supplementary Figure;

Supplementary Figure S1. Enriched terms (p-value<0.05, corrected by Bonferroni method) for molecular function (A) after semantic synthesis by Revigo for cellular component (B) and biological process (C) of differentially expressed genes in HME vs LME in late fattening phases. HME: group of high methane emission cattle (n=5), LME: group of low methane emission cattle (n=5). The intensity of color circles is proportional to the significance of the term while the size is proportional to the number of proteins identified in each term.

Supplementary Tables;

Supplementary Table S1. Values of methane emissions and concentrations of blood metabolites and hormones in HME vs LME cattle during the fattening period. T1: early fattening phases (13 months of age), T2: middle fattening phases (20 months of age), T3: late fattening phases (28 months of age). HME: group of high methane emission cattle (n=6), LME: group of low methane emission cattle (n=6). Values indicate mean. SEM: standard error of the mean. DMI: dry matter intake, BUN: blood urea nitrogen, NEFA: non-esterified fatty acid, ALP: alkaline phosphatase, AST: aspartate aminotransferase, ALT: alanine aminotransferase, γ-GTP: gamma(γ)-glutamyl transferase, LD: lactate dehydrogenase, CK: creatine kinase, BHBA: β-hydroxybutyric acid, IGF-I: insulin-like growth factor 1.

Supplementary Table S2. Concentrations of blood amino acids in HME vs LME cattle during the fattening period.

T1: early fattening phases (13 months of age), T2: middle fattening phases (20 months of age), T3: late fattening phases (28 months of age). HME: group of high methane emission cattle (n=6), LME: group of low methane emission cattle (n=6). Values indicate mean. SEM: standard error of the mean.

**Supplementary Table S3.** **Compositions of rumen fermentations in HME vs LME cattle during the fattening period.** T1: early fattening phases (13 months of age), T2: middle fattening phases (20 months of age), T3: late fattening phases (28 months of age). HME: group of high methane emission cattle (n=6), LME: group of low methane emission cattle (n=6). Values indicate mean. SEM: standard error of the mean.

**Supplementary Table S4.** **Values of methane emissions in Japanese Black cattle during the entire fattening period.** HME: group of high methane emission cattle (n=6), LME: group of low methane emission cattle (n=6).

**Supplementary Table S5. Statistics and mapping results of RNA-seq mapping data in HME vs LME cattle for each fattening phases.** GC: guanine and cytosine, AT: adenine and thymine, Q20: ratio of bases that have phred quality score > 20, Q30: ratio of bases that have phred quality score > 30. T1: early fattening phases (13 months of age), T2: middle fattening phases (20 months of age), T3: late fattening phases (28 months of age). HME: group of high methane emission cattle (n=6), LME: group of low methane emission cattle (n=6).

**Supplementary Table S6.** **Mean of RNA-seq mapping data in HME vs LME cattle in each fattening phases.** GC: guanine and cytosine, AT: adenine and thymine, Q20: ratio of bases that have phred quality score > 20, Q30: ratio of bases that have phred quality score > 30. T1: early fattening phases (13 months of age), T2: middle fattening phases (20 months of age), T3: late fattening phases (28 months of age). HME: group of high methane emission cattle (n=6), LME: group of low methane emission cattle (n=6).

Supplementary Table S7. List of differentially expressed genes in HME vs LME cattle in each fattening phases. T1: early fattening phases (13 months of age), T2: middle fattening phases (20 months of age), T3: late fattening phases (28 months of age).
