# Supplementary figures and images for: Physiological responses and adaptations to high methane production in Japanese Black cattle
Source: Sci Rep. 2022 Jul 1;12:11154. doi: 10.1038/s41598-022-15146-1 (PMC9249741; doi:10.1038/s41598-022-15146-1)

Supplementary Figure S1

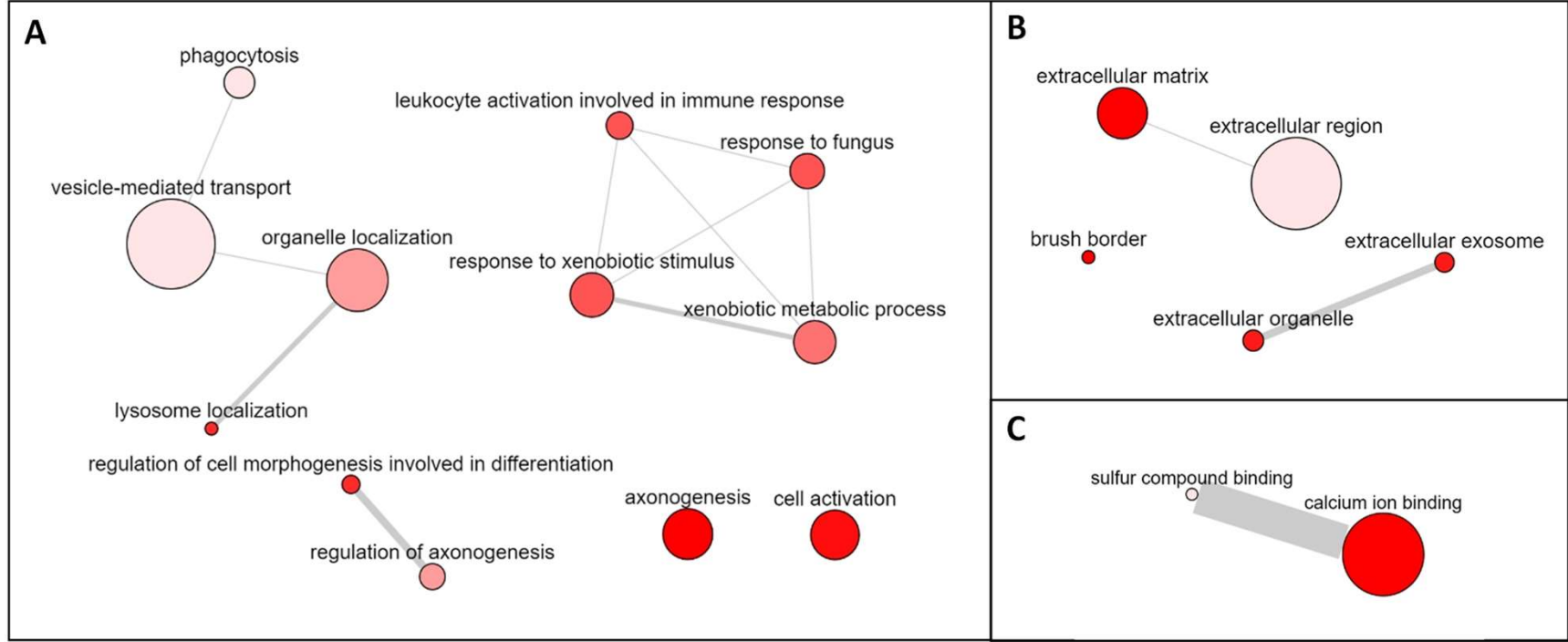

Supplement: Supplementary file 2 — Supplementary Information 2. [file 41598_2022_15146_MOESM2_ESM.pdf]
