# Supplementary material for: Physiological responses and adaptations to high methane production in Japanese Black cattle
Source: Sci Rep. 2022 Jul 1;12:11154. doi: 10.1038/s41598-022-15146-1 (PMC9249741; doi:10.1038/s41598-022-15146-1)
Supplement: Supplementary file 3 — Supplementary Information 3. [file 41598_2022_15146_MOESM3_ESM.pdf]

Supplementary Table S1

Variable	T1				T2				T3			
	HME	LME	SEM	P-value	HME	LME	SEM	P-value	HME	LME	SEM	P-value
**Methane emission**												
Methane emission (L/day)	261.57	220.40	8.39	<0.01	276.94	201.67	13.27	<0.01	282.89	221.15	12.56	<0.01
Methane emisssion / dry matter intake	37.68	31.46	1.20	<0.01	34.86	24.87	1.76	<0.01	39.07	30.20	1.98	0.02
**Blood metabolites**												
Total protein (g/dL)	6.48	6.23	0.11	0.28	6.65	6.37	0.12	0.26	6.63	6.43	0.13	0.47
Albumin (g/dL)	3.60	3.47	0.04	0.14	3.62	3.62	0.04	1.00	3.50	3.60	0.04	0.25
BUN (mg/dL)	14.90	14.15	0.91	0.70	15.48	14.37	0.62	0.40	9.77	9.60	0.44	0.86
Creatinine (mg/dL)	1.09	0.94	0.03	0.01	1.21	1.15	0.03	0.32	1.31	1.18	0.05	0.17
Total cholesterol (mg/dL)	93.33	95.17	4.18	0.84	131.67	130.67	7.60	0.95	96.00	105.83	4.98	0.35
Triglyceride (mg/dL)	13.83	12.00	0.63	0.16	13.33	16.83	1.11	0.12	17.50	17.33	1.19	0.95
NEFA (mEq/L)	0.09	0.07	0.01	0.16	0.09	0.09	0.01	0.66	0.11	0.09	0.00	0.15
Phospholipid (mg/dL)	90.00	89.33	2.83	0.39	121.50	116.67	5.80	0.70	89.67	96.00	3.75	0.42
Glucose (mg/dL)	73.50	69.33	1.50	0.18	66.50	64.83	1.35	0.56	69.00	68.50	1.20	0.85
ALP (U/L)	311.00	353.83	18.09	0.27	199.17	239.00	10.32	0.05	192.00	228.17	7.95	0.01
AST (U/L)	65.33	74.17	1.89	0.01	84.67	83.17	7.80	0.93	64.00	75.83	2.60	0.01
ALT (U/L)	19.17	20.17	0.54	0.38	17.00	17.00	0.54	0.82	19.83	23.17	0.90	0.06
γ-GTP (U/L)	20.17	21.00	0.67	0.56	32.00	29.83	2.95	0.73	20.17	23.33	1.07	0.14
LD (U/L)	928.17	994.83	20.56	0.11	1229.00	1100.67	61.83	0.31	1285.33	1350.17	49.61	0.54
CK (U/L)	144.50	147.00	5.89	0.84	140.17	154.67	11.42	0.55	147.17	133.00	14.28	0.39
Acetate (μmol/L)	33.00	4.67	7.12	0.06	14.83	6.33	3.80	0.31	12.83	5.33	4.46	0.82
BHBA (μmol/L)	822.17	545.83	59.13	0.02	660.33	464.00	36.80	<0.01	606.83	387.50	60.03	0.06
Total ketone body (μmol/L)	855.00	600.17	67.07	0.05	674.50	470.33	39.17	<0.01	619.00	391.83	63.10	0.07
**Hormones**												
Insulin (ng/mL)	3.32	1.32	0.39	<0.01	4.84	3.75	0.73	0.82	5.85	3.94	0.58	0.12
IGF-I (ng/mL)	218.44	223.15	12.17	0.86	143.30	140.81	7.41	0.88	156.24	151.27	11.68	0.84
Cortisol (ng/mL)	6.53	1.99	1.74	0.39	10.50	12.44	4.45	0.31	24.37	24.24	4.95	0.49
