# Supplementary material for: Physiological responses and adaptations to high methane production in Japanese Black cattle
Source: Sci Rep. 2022 Jul 1;12:11154. doi: 10.1038/s41598-022-15146-1 (PMC9249741; doi:10.1038/s41598-022-15146-1)
Supplement: Supplementary file 4 — Supplementary Information 4. [file 41598_2022_15146_MOESM4_ESM.pdf]

Supplementary Table S2

Variable	T1				T2				T3			
	HME	LME	SEM	P-value	HME	LME	SEM	P-value	HME	LME	SEM	P-value
**Blood amino acids (nmol/mL)**												
Taurine	32.42	41.92	3.44	0.18	77.37	84.47	2.64	0.19	36.38	37.72	1.27	0.62
Urea	4721.10	4954.92	262.09	0.68	5431.27	5049.13	227.04	0.39	3500.77	3393.62	163.89	0.76
Aspartic acid	7.28	7.62	0.20	0.43	8.07	8.25	0.39	0.82	7.90	7.62	0.28	0.64
Threonine	72.67	82.18	3.15	0.14	53.43	71.80	3.68	<0.01	61.18	69.47	2.57	0.11
Serine	76.25	87.42	3.07	0.07	80.37	94.12	3.82	0.07	75.72	87.17	2.92	0.07
Glutamic acid	73.33	65.47	3.25	0.24	68.13	63.07	1.93	0.20	76.30	70.80	3.71	0.49
Glutamine	270.47	292.37	9.91	0.29	305.18	310.70	7.36	0.73	335.22	325.77	10.50	0.67
α-Amino adipic acid	14.30	14.55	0.90	0.90	17.55	15.78	0.96	0.38	13.63	11.78	0.56	0.10
Glycine	225.13	249.35	7.80	0.12	192.75	194.78	4.25	0.82	214.80	204.10	7.15	0.48
Alanine	326.28	275.42	21.30	0.25	178.27	198.98	10.12	0.33	218.95	201.08	12.21	0.49
Citrulline	70.50	70.23	3.03	1.00	57.15	64.08	2.48	0.17	57.77	67.42	2.40	0.04
α-Aminobutyric acid	18.05	19.82	1.39	0.56	13.80	13.43	0.72	0.81	8.50	7.72	0.53	0.49
Valine	243.15	294.83	14.67	0.08	228.30	294.38	14.04	<0.01	210.10	259.50	11.05	0.02
Methionine	34.50	36.55	0.94	0.30	28.40	34.32	1.40	0.07	27.27	28.67	0.72	0.35
Cysteine	3.80	3.93	0.21	0.77	4.15	3.50	0.16	0.03	4.78	4.42	0.23	0.44
Isoleucine	122.35	137.47	4.43	0.09	99.15	125.15	6.74	0.05	96.62	109.73	4.92	0.20
Leucine	139.05	167.35	7.30	0.05	134.13	172.08	10.27	0.06	129.17	151.37	7.78	0.16
Tyrosine	63.20	68.50	1.89	0.17	53.85	68.02	3.11	0.01	51.28	56.03	2.29	0.32
Phenylalanine	57.17	67.23	2.21	0.01	61.58	71.70	2.71	0.07	61.90	68.08	1.71	0.07
NH_3	162.33	224.52	16.73	0.08	140.72	134.08	2.11	0.14	187.97	175.30	3.46	0.06
Ornithine	55.67	62.87	2.82	0.22	67.42	77.55	3.81	0.20	49.93	59.90	2.03	<0.01
1-methylhistidine	5.02	4.75	0.26	0.63	7.28	6.48	0.36	0.29	6.72	7.13	0.39	0.62
Histidine	50.23	53.25	1.45	0.32	48.03	52.65	1.33	0.08	49.47	54.57	1.08	<0.01
3-methylhistidine	6.28	5.64	0.21	0.14	8.08	7.48	0.31	0.36	8.60	8.00	0.44	0.53
Lysine	111.62	115.97	3.92	0.60	105.93	130.42	5.94	0.03	98.93	116.48	4.44	0.04
Tryptophan	37.88	39.75	1.41	0.54	44.18	51.05	1.85	0.06	46.98	53.88	1.82	0.04
Arginine	94.98	90.37	3.52	0.54	76.10	79.57	2.93	0.58	79.07	81.28	1.47	0.48
Hydroxyproline	48.53	51.22	1.25	0.30	37.42	34.62	2.61	0.62	34.88	34.37	1.33	0.86
Proline	78.65	82.25	3.76	0.65	77.47	92.47	4.14	0.07	55.38	50.22	3.18	0.18
