# Supplementary material for: Physiological responses and adaptations to high methane production in Japanese Black cattle
Source: Sci Rep. 2022 Jul 1;12:11154. doi: 10.1038/s41598-022-15146-1 (PMC9249741; doi:10.1038/s41598-022-15146-1)
Supplement: Supplementary file 5 — Supplementary Information 5. [file 41598_2022_15146_MOESM5_ESM.pdf]

Supplementary Table S3

Variable	T1				T2				T3			
	HME	LME	SEM	P-value	HME	LME	SEM	P-value	HME	LME	SEM	P-value
pH	6.88	7.08	0.06	0.11	7.00	7.07	0.10	0.72	6.51	6.76	0.07	0.10
NH_3 (mg/dL)	14.88	11.52	0.89	0.05	19.31	13.23	1.22	<0.01	9.45	6.43	0.67	0.02
Total VFA (mmol/dL)	9.98	8.21	0.47	0.05	8.27	8.45	0.62	0.89	11.28	8.94	0.72	0.11
C2 (Acetate) %	61.82	62.34	1.04	0.82	56.93	55.73	1.06	0.60	59.99	59.34	0.50	0.54
C3 (Propionate) %	18.92	21.18	0.65	0.08	22.49	26.25	0.87	0.02	24.13	29.35	1.35	0.05
C4 (Butyrate) %	15.32	12.46	0.93	0.14	15.69	12.98	0.61	0.02	13.32	9.29	0.96	0.03
Others %	3.94	4.02	0.53	0.94	4.89	5.03	0.62	0.91	2.56	2.02	0.15	0.08
C2/C3	3.31	2.95	0.11	0.12	2.57	2.14	0.11	0.05	2.56	2.06	0.15	0.09
