# Supplementary material for: Physiological responses and adaptations to high methane production in Japanese Black cattle
Source: Sci Rep. 2022 Jul 1;12:11154. doi: 10.1038/s41598-022-15146-1 (PMC9249741; doi:10.1038/s41598-022-15146-1)
Supplement: Supplementary file 6 — Supplementary Information 6. [file 41598_2022_15146_MOESM6_ESM.pdf]

Supplementary Table S4

Cattle No.	Methane	Dry matter intake (kg)	Daily gain (kg)	Values of Methane emissions (L/day)		
				Estimated methane emission	Predictive methane emission	Residual methane emission
1	HME	7.84	0.85	251.8	244.7	7.1
2	HME	6.91	0.64	249.4	235.3	14.1
3	HME	7.30	0.78	257.9	239.2	18.7
4	HME	6.97	0.72	263.7	235.9	27.8
5	HME	7.83	0.75	278.8	244.5	34.3
6	HME	7.22	0.77	276.6	238.5	38.1
7	-	7.19	0.71	243.9	238.1	5.8
8	-	7.15	0.66	241.1	237.8	3.3
9	-	7.13	0.75	239.5	237.5	2.0
10	-	6.99	0.70	236.3	236.1	0.2
11	-	6.45	0.60	225.8	230.7	-4.9
12	-	7.15	0.78	231.9	237.7	-5.8
13	-	6.04	0.65	220.6	226.6	-6.0
14	-	7.62	0.84	235.2	242.5	-7.3
15	-	7.26	0.56	226.7	238.9	-12.2
16	LME	7.38	0.81	205.9	240.1	-34.2
17	LME	7.27	0.68	211.2	239.0	-27.8
18	LME	7.97	0.79	228.4	246.0	-17.6
19	LME	7.00	0.83	220.5	236.2	-15.7
20	LME	7.18	0.74	226.5	238.0	-11.5
21	LME	7.97	0.75	237.5	246.0	-8.5
