# Supplementary material for: Physiological responses and adaptations to high methane production in Japanese Black cattle
Source: Sci Rep. 2022 Jul 1;12:11154. doi: 10.1038/s41598-022-15146-1 (PMC9249741; doi:10.1038/s41598-022-15146-1)
Supplement: Supplementary file 7 — Supplementary Information 7. [file 41598_2022_15146_MOESM7_ESM.pdf]

Supplementary Table S5

Sample			Total Bases	Read Count	GC (%)	AT (%)	Q20 (%)	Q30 (%)	Expressed Gene Count
Period	Methane	Cattle No.							
T1	HME	1	4,497,337,696	44,528,096	51.8	48.2	98.3	95.0	15,368
		2	4,263,987,296	42,217,696	47.7	52.3	98.2	94.8	15,434
		3	5,144,782,642	50,938,442	52.2	47.8	98.4	95.2	15,631
		4	5,289,959,840	52,375,840	48.4	51.6	98.3	95.1	15,553
		5	4,830,898,882	47,830,682	52.3	47.7	98.3	95.0	15,068
	LME	6	5,329,484,170	52,767,170	52.5	47.5	98.5	95.5	14,877
		7	5,878,763,580	58,205,580	49.4	50.6	98.3	94.8	16,350
		8	4,792,707,146	47,452,546	50.6	49.5	98.4	95.1	15,930
		9	5,088,895,100	50,385,100	49.6	50.4	98.8	95.9	16,050
		10	4,881,008,214	48,326,814	51.0	49.0	98.6	95.5	15,876
		11	4,535,676,488	44,907,688	48.9	51.1	98.8	95.8	15,779
T2	HME	1	4,792,188,612	47,447,412	49.3	50.7	98.8	96.0	16,183
		2	5,378,922,862	53,256,662	50.4	49.6	98.8	96.1	16,406
		3	4,251,701,050	42,096,050	49.4	50.6	98.8	95.9	15,885
		4	5,593,855,710	55,384,710	49.3	50.7	98.6	95.5	16,124
		5	4,786,869,548	47,394,748	51.5	48.5	98.8	96.1	15,402
		6	4,456,092,124	44,119,724	49.3	50.7	98.8	95.9	15,862
	LME	7	5,137,132,902	50,862,702	49.4	50.6	98.7	95.8	16,300
		8	7,860,394,690	77,825,690	49.2	50.8	98.5	95.2	16,649
		9	4,693,183,766	46,467,166	50.5	49.5	98.7	95.9	15,437
		10	5,013,866,644	49,642,244	51.1	48.9	98.8	96.0	15,318
		11	4,069,150,620	40,288,620	49.3	50.7	98.7	95.9	15,963
T3	HME	1	4,812,245,798	47,645,998	49.2	50.8	98.8	96.1	16,190
		2	4,625,563,660	45,797,660	49.3	50.7	98.7	95.7	15,863
		3	6,184,156,876	61,229,276	49.5	50.5	98.7	95.9	16,644
		4	6,434,995,628	63,712,828	49.3	50.7	98.8	95.9	16,593
		5	4,677,398,476	46,310,876	49.3	50.7	98.7	95.9	15,635
	LME	6	4,337,562,362	42,946,162	48.9	51.1	98.8	96.0	15,772
		7	4,378,407,166	43,350,566	49.0	51.0	98.7	95.7	15,688
		8	6,904,254,152	68,358,952	49.8	50.2	98.5	95.2	16,619
		9	4,798,324,564	47,508,164	49.2	50.8	98.8	95.9	15,852
		10	4,797,886,426	47,503,826	48.3	51.7	98.8	96.0	16,182
