# Supplementary material for: Physiological responses and adaptations to high methane production in Japanese Black cattle
Source: Sci Rep. 2022 Jul 1;12:11154. doi: 10.1038/s41598-022-15146-1 (PMC9249741; doi:10.1038/s41598-022-15146-1)
Supplement: Supplementary file 8 — Supplementary Information 8. [file 41598_2022_15146_MOESM8_ESM.pdf]

Supplementary Table S6

Sample			Total Bases	Read Count	GC (%)	AT (%)	Q20 (%)	Q30 (%)	Expressed Gene Count
**Period**	**Methane**	**N**							
T1	HME	5	4,805,393,271	47,578,151	50.5	49.5	98.3	95.0	15,411
	LME	6	5,084,422,450	50,340,816	50.3	49.7	98.5	95.4	15,810
T2	HME	6	4,876,604,984	48,283,218	49.9	50.1	98.8	95.9	15,977
	LME	5	5,354,745,724	53,017,284	49.9	50.1	98.7	95.8	15,933
T3	HME	5	5,346,872,088	52,939,328	49.3	50.7	98.7	95.9	16,116
	LME	5	5,043,286,934	49,933,534	49.0	51.0	98.7	95.8	16,085
