# Supplementary material for: Physiological responses and adaptations to high methane production in Japanese Black cattle
Source: Sci Rep. 2022 Jul 1;12:11154. doi: 10.1038/s41598-022-15146-1 (PMC9249741; doi:10.1038/s41598-022-15146-1)
Supplement: Supplementary file 9 — Supplementary Information 9. [file 41598_2022_15146_MOESM9_ESM.pdf]

Supplementary Table S7

Period	Gene type	Gene ensembl ID	Gene symbol	Description	BaseMean	log2FoldChange	Style	P-value	Padj
T1	mRNA	ENSBTAG00000049359	NA	NA	68.37	-23.34	DOWN	<0.01	<0.01
	mRNA	ENSBTAG00000003152	IFI27	Interferon alpha inducible protein 27	28315.56	2.50	Up	<0.01	<0.01
	mRNA	ENSBTAG00000006185	SPC24	SPC24 component of NDC80 kinetochore complex	337.14	-1.81	DOWN	<0.01	0.04
	mRNA	ENSBTAG00000006440	IGDCC4	Immunoglobulin superfamily DCC subclass member 4	77.80	1.35	Up	<0.01	0.03
	mRNA	ENSBTAG00000006731	SLC7A5	Solute carrier family 7 member 5	309.04	-2.98	DOWN	<0.01	<0.01
	mRNA	ENSBTAG00000011423	SERPINI2	Serpin family I member 2	117.25	4.78	Up	<0.01	0.04
T2	mRNA	ENSBTAG00000015919	PARM1	Prostate androgen-regulated mucin-like protein 1	101.56	1.88	Up	<0.01	<0.01
	mRNA	ENSBTAG00000016711	PPIF	Peptidylprolyl isomerase F	5879.91	-1.09	DOWN	<0.01	0.05
	mRNA	ENSBTAG00000019947	SYT7	Synaptotagmin 7	262.61	-1.05	DOWN	<0.01	0.04
	mRNA	ENSBTAG00000038112	NA	NA	65.63	2.56	Up	<0.01	0.02
	mRNA	ENSBTAG00000049496	NA	NA	184.22	4.98	Up	<0.01	<0.01
	mRNA	ENSBTAG00000051211	DUSP8	Dual specificity phosphatase 8	147.96	-1.45	DOWN	<0.01	0.05
	mRNA	ENSBTAG00000005170	ADGRG5	Adhesion G protein-coupled receptor G5	55.81	-1.02	DOWN	<0.01	0.05
	mRNA	ENSBTAG00000001117	ANKRD50	Ankyrin repeat domain 50	142.22	1.09	Up	<0.01	0.02
	mRNA	ENSBTAG00000043584	ATP6	ATP synthase F0 subunit 6	255857.34	-1.54	DOWN	<0.01	0.02
	mRNA	ENSBTAG00000006595	C25H16orf71	Chromosome 25 open reading frame, human c16orf71	67.27	1.02	Up	<0.01	0.02
	mRNA	ENSBTAG00000010151	CA5A	Carbonic anhydrase 5A	645.21	-1.29	DOWN	<0.01	<0.01
	mRNA	ENSBTAG00000007312	CD209	CD209 molecule	153.12	-1.29	DOWN	<0.01	<0.01
	mRNA	ENSBTAG00000019033	CD84	Cluster of differentiation 84	91.62	-1.02	DOWN	<0.01	0.02
	mRNA	ENSBTAG00000014546	CLEC7A	C-type lectin domain family 7 member A	62.57	-1.28	DOWN	<0.01	0.03
	mRNA	ENSBTAG00000013802	DAB1	Reelin adaptor protein	328.58	1.15	Up	<0.01	<0.01
	mRNA	ENSBTAG00000014306	DOP1B	Dopey family member 2	161.68	-1.28	DOWN	<0.01	<0.01
	mRNA	ENSBTAG00000004014	FBLN2	Fibulin 2	336.59	1.13	Up	<0.01	0.01
	mRNA	ENSBTAG00000051210	GSTP1	Glutathione s-transferase Pi 1	3474.35	-1.01	DOWN	<0.01	<0.01
	mRNA	ENSBTAG00000015235	HMCN1	Hemicentin 1	52.92	2.12	Up	<0.01	<0.01
	mRNA	ENSBTAG00000007881	IFIT1	Interferon-induced protein with tetratricopeptide repeats 1	193.56	-1.08	DOWN	<0.01	0.01
	mRNA	ENSBTAG00000048470	IFITM1	Interferon induced transmembrane protein 1	895.80	-1.13	DOWN	<0.01	<0.01
	mRNA	ENSBTAG00000018156	KRTCAP3	Keratinocyte associated protein 3	348.43	-2.08	DOWN	<0.01	<0.01
	mRNA	ENSBTAG00000005910	LEPR	Leptin receptor	369.92	1.03	Up	<0.01	<0.01
	mRNA	ENSBTAG00000025258	LOC515676	Keratin-associated protein 10-6	727.85	-1.49	DOWN	<0.01	<0.01
	mRNA	ENSBTAG00000017040	LY6E	Lymphocyte antigen 6 complex, locus E	1171.39	-1.01	DOWN	<0.01	<0.01
	mRNA	ENSBTAG00000001961	MAP1B	Microtubule associated protein 1B	102.43	1.69	Up	<0.01	0.01
	mRNA	ENSBTAG00000018473	MARCO	Macrophage receptor with collagenous structure	8503.36	-1.03	DOWN	<0.01	0.01
T3	mRNA	ENSBTAG00000039803	MYO7B	Myosin viiB	53.27	4.61	Up	<0.01	0.04
	mRNA	ENSBTAG00000043551	NA	NA	64.67	-1.90	DOWN	<0.01	<0.01
	mRNA	ENSBTAG00000043566	NA	NA	85.70	-1.73	DOWN	<0.01	<0.01
	mRNA	ENSBTAG00000043554	NA	NA	91.19	-1.55	DOWN	<0.01	<0.01
	mRNA	ENSBTAG00000043544	NA	NA	101.94	-1.74	DOWN	<0.01	<0.01
	mRNA	ENSBTAG00000049825	NA	NA	109.33	-1.19	DOWN	<0.01	0.01
	mRNA	ENSBTAG00000043552	NA	NA	160.23	-1.87	DOWN	<0.01	<0.01
	mRNA	ENSBTAG00000043576	NA	NA	166.06	-1.48	DOWN	<0.01	<0.01
	mRNA	ENSBTAG00000052521	NA	NA	322.68	1.71	Up	<0.01	0.01
	mRNA	ENSBTAG00000048135	NA	NA	851.54	1.17	Up	<0.01	<0.01
	mRNA	ENSBTAG00000048980	NA	NA	2317.01	-1.51	DOWN	<0.01	<0.01
	mRNA	ENSBTAG00000007247	NUF2	NDC80 kinetochore complex component	68.07	-1.50	DOWN	<0.01	<0.01
	mRNA	ENSBTAG00000015919	PARM1	Prostate androgen-regulated mucin-like protein 1	67.83	1.18	Up	<0.01	0.04
	mRNA	ENSBTAG00000012991	PRUNE2	Prune homolog 2	184.82	-1.81	DOWN	<0.01	0.03
	mRNA	ENSBTAG00000019630	RGL1	Ral guanine nucleotide dissociation stimulator like 1	657.90	-1.02	DOWN	<0.01	0.04
	mRNA	ENSBTAG00000013929	RRAD	Ras related glycolysis inhibitor and calcium channel regulator	79.15	1.53	Up	<0.01	0.02
	mRNA	ENSBTAG00000012638	S100A12	S100 calcium binding protein a12	333.80	-1.15	DOWN	<0.01	0.05
	mRNA	ENSBTAG00000011423	SERPINI2	Serpin family I member 2	320.67	5.01	Up	<0.01	<0.01
	mRNA	ENSBTAG00000006731	SLC7A5	Solute carrier family 7 member 5	132.61	-2.63	DOWN	<0.01	<0.01
	mRNA	ENSBTAG00000017746	SLIT3	Slit guidance ligand 3	57.98	1.57	Up	<0.01	0.04
	mRNA	ENSBTAG00000005623	SOAT2	Sterol o-acyltransferase 2	363.21	-1.24	DOWN	<0.01	0.03
	mRNA	ENSBTAG00000013185	TIMD4	T-cell immunoglobulin and mucin domain containing 4	1183.45	-1.09	DOWN	<0.01	<0.01
	mRNA	ENSBTAG00000016357	VNN2	Vanin 2	165.78	-1.10	DOWN	<0.01	0.01
